# Upregulation of p27 and its inhibition of CDK2/cyclin E activity following DNA damage by a novel platinum agent are dependent on the expression of p21

**DOI:** 10.1038/sj.bjc.6603448

**Published:** 2006-11-07

**Authors:** G He, J Kuang, Z Huang, J Koomen, R Kobayashi, A R Khokhar, Z H Siddik

**Affiliations:** 1 Department of Experimental Therapeutics, Unit 353, The University of Texas MD Anderson Cancer Center, 1515 Holcombe Blvd, Houston, TX 77030, USA; 2 Department of Molecular Pathology, The University of Texas MD Anderson Cancer Center, Houston, TX 77030, USA

**Keywords:** DNA damage, G1-phase cyclin-dependent kinase, platinum complex, p27 regulation

## Abstract

The cisplatin analogue 1*R*,2*R*-diaminocyclohexane(*trans*-diacetato)(dichloro)platinum^IV^ (DAP) is a DNA-damaging agent that will be entering clinical trials for its potent cytotoxic effects against cisplatin-resistant tumour cells. This cytotoxicity may reside in its ability to selectively activate G1-phase checkpoint response by inhibiting CDKs via the p53/p21 pathway. We have now evaluated the role of another CDK inhibitor p27 as a contributor to DAP-mediated inhibition of G1-phase CDK2 activity. Our studies in ovarian A2780 tumour cells demonstrate that p27 levels induced by DAP are comparable to or greater than those seen for p21. The induction of p27 is not through a transcriptional mechanism, but rather is due to a four-fold increase in protein stabilisation through a mechanism dependent on p21. Moreover, DAP-induced p21 promoted the selective increase of p27 in the CDK2 complex, but not in CDK4 complex, and this selective increase contributed to inhibition of the CDK2 kinase activity. The inhibited complex contained either p27 or p21, but not both, with the relative levels of cyclin E associated with p27 and p21 indicating that about 25% of the inhibition of CDK2 activity was due to p27 and 75% due to p21. This study provides the first evidence that p27 upregulation is directly attributable to activation of the p53/p21 pathway by a DNA-damaging agent, and promulgates p53/p21/p27 axis as a significant component of checkpoint response.

A major limitation in the therapy of cancer is the onset of drug resistance. In this regard, the cisplatin analogue 1*R*,2*R*-diaminocyclohexane(*trans*-diacetato)(dichloro)platinum^IV^ (DAP) will undergo clinical trials as a therapeutically promising DNA-damaging agent owing to its ability to circumvent cisplatin resistance (Kido *et al*, 1993; Siddik *et al*, 1994; Mujoo *et al*, 2003, 2005), but the precise mechanism of how DAP accomplishes this remains to be resolved. However, its ability to induce G1-phase cell cycle arrest may be a significant therapeutic feature, and this is supported by the recent demonstration that selective small molecule inhibitors of the G1-phase CDK4/cyclin D or CDK2/cyclin E complex are cytotoxic (Senderowicz, 2003). Our previous investigations using A2780 ovarian cancer cell line as a model system have established that DAP specifically and simultaneously inhibited activities of these two G1-phase kinases (Kuang *et al*, 2001; He *et al*, 2005). To affect inhibition, activation of the p53-p21 pathway was essential, and this resulted in the binding of the induced p21 to both CDK complexes. Thus, it is likely that these inhibitory effects of DAP on G1-phase CDKs induce drug-dependent cytotoxicity and contribute to its ability to circumvent cisplatin resistance. Indeed, deletion of p21 in colon tumour cells prevented DAP-mediated cell cycle effects (Kuang *et al*, 2001; He *et al*, 2005), which generally precede cell death. This is also consistent with our report that inhibiting p53 function leads to DAP resistance (Hagopian *et al*, 1999). Moreover, in cisplatin-resistant tumour cells, p21 is induced by DAP but not cisplatin (Mujoo *et al*, 2003, 2005), and this supports the premise that inhibition of CDKs may be necessary for DAP to circumvent cisplatin resistance. Therefore, it becomes critically important to fully understand the mechanism of G1-phase checkpoint response induced by DAP.

The role of p21 in DAP-induced G1 checkpoint response is in keeping with numerous reports that have demonstrated p21 to be a critical mediator of G1-phase cell cycle arrest following exposure to DNA-damaging agents (Macleod *et al*, 1995; Waldman *et al*, 1995). However, p27 upregulation can also occur following DNA damage with UV radiation or antitumour drugs, including cisplatin (Petrocelli and Slingerland, 2000; Zhou *et al*, 2004), and p27 has the potential to inhibit CDK2/cyclin E and, thereby induce G1-phase arrest, regulate cell growth and, as a result, influence outcome of therapy with antitumour agents (Balasubramanian *et al*, 1999; Qin and Ng, 2002), which could possibly include DAP. In contrast, both p21 and p27 also have counterintuitive, but essential roles in the assembly of the active CDK4 complex (Cheng *et al*, 1999). Despite such similarities in functions, it is noteworthy that underlying mechanisms regulating p21 and p27 induction are distinct, with regulation occurring predominantly at the transcriptional level for p21 and at the post-translational level for p27. However, the induction of p27 is poorly understood. On the other hand, some evidence exists to indicate that degradation of p27 is mediated via the proteasome pathway, which requires initial phosphorylation of p27 at Thr-187 for recognition by the E3 ubiquitin ligase SCF/Skp2 complex (Bloom and Pagano, 2003). Therefore, p27 levels may be induced by mechanisms that inhibit p27 degradation, such as that observed with proteasome inhibitors MG132 and lovastatin (Lee and Goldberg, 1998; Rao *et al*, 1999), or following downregulation of Skp2 by siRNA (Jiang *et al*, 2005). There are scant reports to suggest that p21 may also increase p27 levels (Liu *et al*, 2000; Steinman *et al*, 2001; Munoz-Alonso *et al*, 2005), but other studies contradict this, as no increases in p27 were demonstrated when p21 was induced either ectopically (Yamamoto *et al*, 1999) or following treatment with UCN-01 (Facchinetti *et al*, 2004).

We have previously observed that p27 is bound to the CDK2 complex in lysates from cells exposed to DAP (He *et al*, 2005), but the underlying mechanism or significance of this binding was not delineated. As checkpoint response to DAP-induced DNA damage through CDK inhibition may trigger cytotoxic effects, it is important to know the consequence of the binding of p27 to CDK on its kinase activity. In the present study, therefore, we have investigated the effect of DAP on p27 levels, and whether the p27 participates in inhibiting CDK2/cyclin E. Our results indicate that p27 induction by DAP is substantial and comparable to that seen for p21. More importantly, we demonstrate for the first time that DAP as a DNA-damaging agent induces p27 in a p21-dependent manner, and that p27 contributes significantly in inhibiting CDK2/cyclin E activity in ovarian A2780 tumour cells.

## EXPERIMENTAL PROCEDURES

### Chemicals, tissue culture and cytotoxicity assessment

DAP was synthesised as reported previously (Ali Sr. *et al*, 2000), and a stock solution was prepared in deionised water. Cycloheximide and MG132 were purchased from Sigma Chemical Company (St Louis, MO, USA). A2780 cells were maintained as monolayer cultures in RPMI 1640 medium supplemented with 10% foetal bovine serum. As described previously (Hagopian *et al*, 1999; Kuang *et al*, 2001), the antiproliferative effect, viability or apoptosis was assessed with a range of DAP concentrations using the MTT assay after 5 days of drug exposure, trypan blue exclusion after 4 days of drug exposure or Annexin V assay after 3 days of drug exposure, respectively. These were optimal times for assessing these effects. The IC_50_ (drug concentration inducing 50% cytotoxicity) was assessed from fitting a sigmoidal curve to the data using the SigmaPlot 2000 software program, and the data are presented as mean±s.e. (*N*=7 separate data sets). For biochemical and molecular assays, cells (1–1.3 × 10^6^) were plated in 100 mm culture dishes and cultured for at least 24 h before the cultures were changed into fresh medium containing 0.6 *μ*M DAP. The cells were further cultured for indicated times before they were extracted for total proteins by using procedures described previously (Kuang *et al*, 2001).

### Plasmids and transfection

His-p21 and His-p27 vectors and human p21 or p27 cDNA were kindly provided by Dr Mong-Hong Lee. The cDNA was used as the template for PCR amplification. For the myc-p21 vector, the forward primer was 5′-ccggaattcggatcccgatgtcagaaccggctggg-3′, which contained an *Eco*RI site, whereas the reverse primer 5′-ccgctcgagttagggcttcctcttggagaa-3′ contained an *Xho*I site. For Myc-p27, the forward primer 5′-ccggaattcggatcccgatgtcaaacgtgcgagtgtc-3′ and the reverse primer 5′-ccgctcgagttacgtttgacgtcttctgagg-3′ contained identical restriction sites. After the PCR product was digested with both *Eco*RI and *Xho*I enzymes, it was subcloned into pCMV-Myc vector (Clontech, Mountain View, CA, USA).

Myc-p21, Myc-p27 or empty vector was transfected into A2780 cells with LipofectAMINE 2000 (LF2000, catalogue no. 11668-019) from Invitrogen (Carlsbad, CA, USA), using the manufacturer's instructions (version: 07/09/2004). Cells were exposed to the transfection mixture containing 4 *μ*l of LF2000 and 2 *μ*g of plasmids for 5 h, washed and then replenished with normal media. Cells were re-incubated and collected after 24 h to prepare lysates, as described before (Kuang *et al*, 2001).

### Northern blot analysis

The procedure has been previously reported by us (He *et al*, 2005). Briefly, total RNA was extracted from A2780 cells and then separated by electrophoresis through a 0.8% agarose gel containing 1.5% (v v^−1^) formaldehyde. RNA was transferred to nylon membranes, hybridised with ^32^P-labelled p27 or glyceraldehyde-3-phosphate dehydrogenase cDNA at 42°C for 18 h, and washed with 0.1 × standard saline citrate/0.1% sodium dodecyl sulphate (SDS) for at least 30 min. Blots were then exposed to X-ray film for 6–16 h.

### RNA interference

Both p21 siRNA duplexes (catalogue ID 1621), which targets a proprietary segment in Exon 3 of p21 mRNA, and control siRNA (catalogue ID 4611) were purchased from Ambion (Austin, TX, USA). A2780 cells were transfected with 100 nM siRNA using LipofectAMINE 2000 (LF2000), as described above. The depletion of p21 protein levels by siRNA is maintained for at least 2 days, and all experiments were completed within this time. Cells were incubated with the transfection mixture for 5 h, washed, re-incubated with complete media for 1 h, and then exposed to DAP (0.6 *μ*M final concentration). Cells were collected at 30 h after initiating DAP treatment, and lysates prepared, as described above.

### Immunoprecipitation, immunoblotting and kinase assay

Immunoprecipitation was performed as described previously (Kuang *et al*, 2001). The antibodies used to immunoprecipitate CDK4 (sc-260), CDK2 (sc-163), cyclin D (sc-718), p21 (sc-397) and p27 (sc-528) were purchased from Santa Cruz Biotechnology (Santa Cruz, CA, USA). The antibody to immunoprecipitate cyclin E (PC438) was purchased from Oncogene Research Products (Boston, MA, USA).

Immunoblotting for individual proteins was performed as described previously (Kuang *et al*, 2001). Primary antibodies used to immunoblot CDK4 (MS-299), CDK2 (MS-617) and p27 (MS-256) were purchased from Neomarkers Inc. (Fremont, CA, USA). Those used to immunoblot cyclin D1 (sc-20044), cyclin E (sc-247) and p21 (sc-6246) were obtained from Santa Cruz Biotechnology, whereas the antibody for *β*-actin (A5060) was purchased from Sigma Chemical Company (St Louis, MO, USA). The horseradish peroxidase-conjugated secondary antibody was acquired from Amersham Pharmacia (Piscataway, NJ, USA). The protein bands on immunoblots were visualised by ECL detection (Amersham Pharmacia) using different lengths of exposures, with exposures that gave linear or near-linear range of signals selected. To quantify signals on immunoblots, the developed films were scanned and band densities analysed with NIH Image software (version 1.62).

Simultaneous quantification of p21 and p27 on immunoblots was validated using calibrated amounts of purified standard proteins. Recombinant His-tagged p21 and p27 were produced in *E. coli*, purified by affinity chromatography on Ni-NTA agarose column according to the manufacturer's instruction (Qiagen Inc., Valencia, CA, USA), and quantified using bovine serum albumin (Sigma) as the standard. Recombinant p21 and p27 proteins of known molar ratios were then simultaneously separated by SDS–PAGE, transblotted to nitrocellulose membrane and immunoblotted with a combination of p21 and p27 monoclonal antibodies. For maximal primary antibody bindings, the blots were incubated with 2 *μ*g ml^−1^ of each of the antibodies at 4°C for at least 14 h. This antibody concentration was 5–10 times higher than the concentration used for our standard immunoblots, and the 14-h incubation time was 4–7 times longer than the standard procedure. After exposure to the primary antibody binding, the blots were processed as for the standard immunoblots. Finally, the simultaneously developed p21 and p27 bands on the same immunoblot were quantified and their relative intensities determined. Comparison of the relative protein intensities by immunoblot analysis against relative Coomassie blue staining intensities allowed an assessment of whether it was valid to determine p21 and p27 simultaneously on the same immunoblot.

Kinase assay to assess CDK2 complex activity was performed as described before (Kuang *et al*, 2001).

### Gel filtration chromatography

All gel filtration chromatographies were performed at 4°C on a 40-ml Ultrogel AcA34 column (LKB, Houston, TX, USA) with a buffer containing 80 mM *β*-glycerophosphate, 20 mM EGTA, 150 mM NaCl, 15 mM MgCl_2_ and 1 mM diothiothreitol. The molecular weight standards used to calibrate the column were purchased from Bio-rad (Hercules, CA, USA) and Amersham (Piscataway, NJ, USA). For each gel filtration, 0.5 ml of the standards or crude cell lysates supplemented with 0.5 mg of purified glutathione *S*-transferase were loaded onto the column and chromatographed at a flow rate of ∼0.3 ml min^−1^ with collection of 1-ml fractions. Immediately after chromatography, 40 *μ*l aliquots were taken from fractions 19 to 35 and separated by 12.5% SDS–PAGE. The membranes were then immunoblotted with anti-p27 antibody using the standard procedure. To normalise the signals from different chromatographies for comparison, the highest signal for each protein from each chromatography was arbitrarily defined as 100 and other signals were normalised to this.

### Immunoprecipitation for protein identification by mass spectrometry

Fifteen millilitres of crude cell lysates (∼10 mg protein ml^−1^) were prepared from untreated or DAP-treated (0.6 *μ*M × 30 h) A2780 cells as described above, and pre-absorbed for 4 h at 4°C with 200 *μ*l of protein-A agarose (Sigma). To isolate the p27-containing complex, the pre-cleared cell lysates were incubated overnight at 4°C with 20 *μ*g of polyclonal anti-p27 antibodies or rabbit IgG (Sigma) that were covalently linked to 30 *μ*l of Affi-prep protein A beads (Bio-Rad) through dimethylpimelimidate catalysed reaction (Schneider *et al*, 1982). The beads were then washed six times for 3 h each time with 0.5% NP-40, 150 mM NaCl, 2 mM EDTA, 2 mM EGTA, 5 mM sodium *β*-glycerophosphate, 10 mM NaF, 10% glycerol, 1 mM DTT and 1 mM PMSF in 50 mM Tris-HCl (pH 7.4), and the precipitated proteins were eluted with SDS–PAGE sample buffer and resolved by 12.5% SDS–PAGE. Following electrophoresis, each gel slab was fixed overnight in 40% ethanol and 10% acetic acid and washed first for 10 min with 40% ethanol and then for another 15 min with deionised water. The gel was next sensitised by a 1-min incubation in 0.02% sodium thiosulphate followed by two 1-min rinses with distilled water. After rinsing, the gel was submerged in 0.1% silver nitrate for 30 min and developed in 0.04% formalin in 3% sodium carbonate to desired intensities. The development was stopped by addition of one-tenth volume of 2.3 M citric acid to the developer and incubation of the gel in this solution for 15 min. The gel was then rinsed three times for 10 min each time with distilled water and stored in distilled water at 4°C (Morrissey, 1981; Shevchenko *et al*, 1996).

### Protein identification by mass spectrometry

Polypeptide bands of interest were excised from the silver-stained gels and de-stained with the Invitrogen Silver Quest kit (Carlsbad, CA, USA). After de-staining, the bands were washed five times with distilled water and twice with 25 mM ammonium bicarbonate in 50% acetonitrile. The bands were then digested for 20 h with trypsin (Promega, Madison, WI, USA) in 25 mM ammonium bicarbonate, and the peptide fragments were extracted twice with 0.01% trifluoroacetic acid in 50% acetonitrile and concentrated to less than 10 *μ*l by centrifugation in a Speedvac (Thermo Savant, Holbrook, NY, USA). The peptides were purified by HPLC on C-18 Ziptip (Millipore, Bedford, MA, USA) by using 2% acetonitrile/0.1% formic acid to wash and 70% acetonitrile/0.1% formic acid to elute the peptides. After the acetonitrile was removed from the collected samples by centrifugation in a Speedvac for 2–3 min, peptide mass fingerprinting (PMF) was performed on a Voyager DE-STR MALDI TOF instrument (Applied Biosystems, Framingham, MA, USA) in positive ion and reflected mode. Dried droplet deposits of *α*-cyano-4-hydroxycinnamic acid were prepared by mixing the peptide solutions 1 : 1 with 10 mg ml^−1^ matrix in methanol. Database searching was performed by using Mascot (http://www.matrixscience.com) and Protein Prospector (http://prospector.ucsf.edu). To validate PMF results, peptides were sequenced by using an LCQ Deca XP electrospray quadrupole ion trap mass spectrometer (Thermo Finnigan, San Jose, CA, USA) coupled to an HP 1090 liquid chromatography (Agilent, Palo Alto, CA, USA). The solvent system for the HPLC included solution A (2% acetonitrile/0.01% trifluoroacetic acid) and solution B (98% acetonitrile/0.01% trifluoroacetic acid). A 40-min linear gradient of 5–60% B was run to elute the peptides from a 0.3-mm C-18 column (Vydac, Hesperia, CA, USA). Data-dependent acquisition was performed for 60 min by using two MS-MS steps after each MS1. Database search with the MS-MS data was performed with Sonar (Genomic Solutions, Ann Arbor, MI, USA).

## RESULTS

### Cellular effects of DAP

The cellular effects of DAP were assessed against A2780 cells using three different standard assays, namely mitochondrial reduction of the MTT dye, cellular membrane integrity by trypan blue exclusion and Annexin V apoptotic assay. Interestingly, the IC_50_ determined with each of these assays were similar, with 0.22±0.05 *μ*M obtained in the MTT assay, 0.26±0.03 *μ*M with trypan blue and 0.31±0.09 *μ*M by Annexin V. Therefore, the 0.6 *μ*M drug concentration used predominantly in the following studies was about 2–3 × IC_50_ level. This exposure level has been previously demonstrated by us to preferentially induce G1-phase arrest in A2780 cells (Kuang *et al*, 2001).

### DAP treatment upregulates the expression of p27

**Figure 1:**
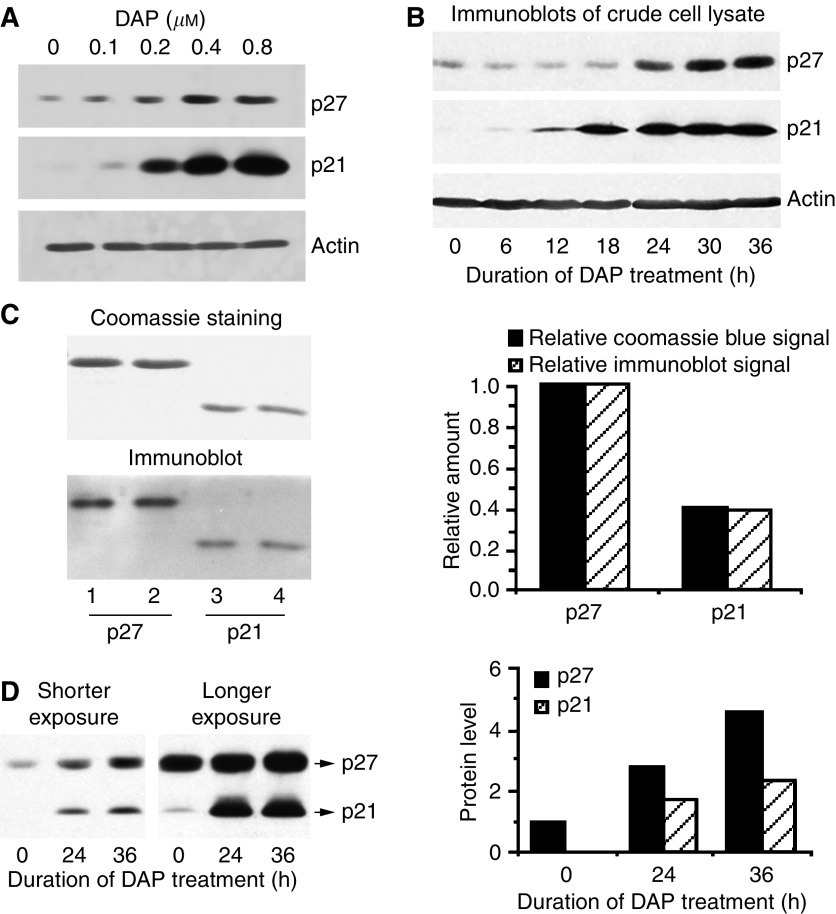
DAP upregulates the expression of p21 and p27. (**A**) A2780 cells were incubated with different concentrations of DAP for 30 h. Crude cell lysates were prepared and immunoblotted individually with antibody specific to p21, p27 or actin. (**B**) Total proteins extracted from A2780 cells treated with DAP (0.6 *μ*M) for the indicated lengths of time were immunoblotted individually with antibody that recognises each of the indicated protein. (**C**) Purified His-tagged p27 and p21 in about a 2 : 1 ratio were separated by SDS–PAGE and stained with Coomassie blue (left panel). The same samples were also diluted 1 : 1000 and simultaneously immunoblotted with anti-p27 and anti-p21 antibodies under saturating immunoblotting conditions. The Coomassie blue staining and immunoblot signals were then quantified by densitometry, and the mean levels normalised to the signal for p27 were plotted (right panel). (**D**) Total proteins extracted from A2780 cells treated with DAP (0.6 *μ*M) for 0, 24 and 36 h were simultaneously immunoblotted with anti-p21 and anti-p27 antibodies (left panel). The signals were quantified, normalised against the level of p27 at 0 h and plotted (right panel).

To explore whether p27 plays a role in DAP-induced inhibition of G1-phase progression, we treated A2780 cells with different concentrations of DAP (0.2–0.8 *μ*M) for 30 h and determined whether this treatment increased expression of p27. Immunoblotting of crude cell lysates showed that p21 was induced in a dose-dependent manner (Figure 1A). p27 was also induced in parallel, but the induction did not increase further above 0.4 *μ*M. To establish a temporal profile, we treated A2780 cells with 0.6 *μ*M of DAP for up to 36 h and determined changes in the levels of p21 and p27. Immunoblotting of crude cell lysate following DAP treatment showed that p21 was induced at 12 h, reaching maximum levels during the next 18–36 h time period. Similarly, p27 was induced at 24 h and reached plateau levels by 30 h (Figure 1B). Together, these results demonstrate that DAP treatment induces both p21 and p27. However, it is apparent that induction of p27 lags behind that of p21, suggesting the possibility that p21 may act as a positive regulator of p27.

Although both p21 and p27 levels increase during DAP treatment, it is difficult to assess the quantitative increase of one relative to the other. This is inherent to the immunoblot technique as densitometric quantification is influenced by several factors, including protein loading, interaction with the antibody, ECL reaction and time of film development. To accurately quantify relative levels of each inhibitor in this study, immunoblots were developed simultaneously with both anti-p21 and anti-p27 monoclonal antibodies under saturating conditions (see Experimental Procedures). Under such conditions, if the monoclonal antibodies recognise p21 and p27 with similar efficiencies, then Coomassie blue staining and immunoblot signals of purified inhibitor proteins should give similar p27 : p21 ratios for the band intensities. The results from duplicate protein loading are presented in Figure 1C, and demonstrate that the relative levels of p27 (lanes 1 and 2) and p21 (lanes 3 and 4) are similar with either Coomassie staining or immunoblot. This validation of the approach with purified proteins enabled crude lysates from DAP-treated cells to be immunoblotted for p21 and p27 simultaneously in an identical manner. The results demonstrate that although the level of p27 greatly exceeded that of p21 in untreated cells, the difference became less pronounced (⩽2-fold) after 24–36 h of DAP treatment (Figure 1D). It is notable that the absolute amount of p27 induced is similar to or greater than p21, suggesting the possibility that as with p21, p27 also contributes to DAP-mediated inhibition of CDK4 and CDK2 activities.

### DAP upregulates p27 by inhibiting its degradation

**Figure 2:**
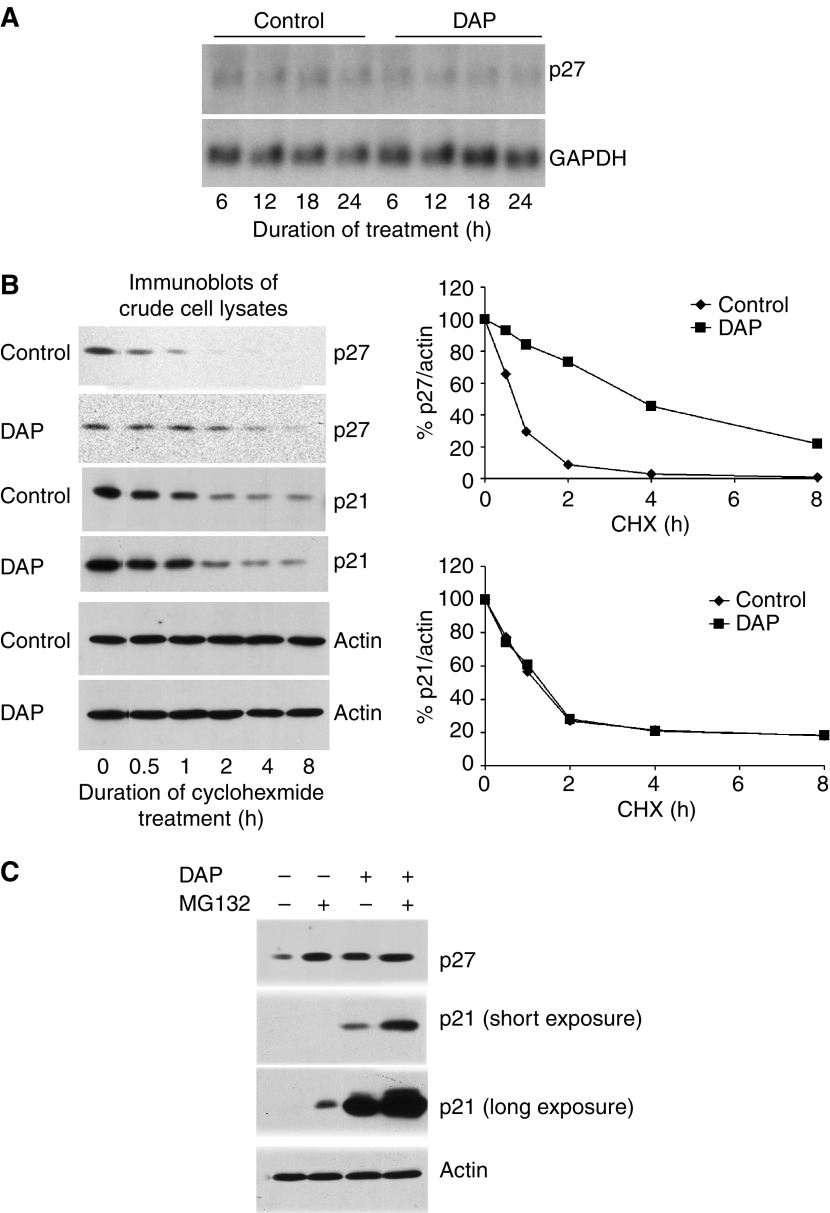
DAP-induced p21 increases p27 by inhibiting p27 degradation. (**A**) Total RNA extracted from A2780 cells that had been treated with 0.6 *μ*M DAP for the indicated length of time was hybridised with ^32^P-labelled p27 and GAPDH cDNAs. (**B**) Control or 30-h DAP-treated A2780 cells were incubated with PBS or 4 *μ*M cyclohexmide for 8 h. Cells were collected at the indicated time points (the 30-h time point after 0.6 *μ*M DAP treatment was arbitrarily set as time zero). Crude cell lysates were then separated with SDS–PAGE and immunoblotted with antibodies specific for p21, p27 and actin, respectively (left panel). The bands were quantified by densitometry, normalised to actin band intensity and plotted against time, with signal at time zero set at 100% (right panel). (**C**) Control or DAP-treated (0.6 *μ*M; 30 h) A2780 cells were incubated with vehicle or 10 *μ*M MG132 for 6 h. Crude cell lysates were then separated with SDS–PAGE and immunoblotted with antibodies specific for p21, p27 and actin, respectively.

The previous section demonstrated that DAP treatment leads to elevation in p27 levels. To explain this elevation, we first examined the effect of DAP on mRNA levels of p27 by Northern blot analysis. However, there was no change in the message, ruling out a transcriptional mechanism for p27 upregulation (Figure 2A). To examine inhibition of p27 degradation as a possible cause, we incubated control or DAP-treated A2780 cells with the protein synthesis inhibitor cycloheximide and determined the half-life of p27 in parallel with p21. Immunoblotting of crude lysates from cells collected at different time points during cycloheximide treatment, followed by quantification of bands on immunoblots and then normalising to *β*-actin, showed that the half-life of p27 increased from 0.75 to 3 h in response to DAP treatment (Figure 2B). In contrast, the half-life of p21 of about 1.25 h was unaffected by DAP. These results indicate that DAP-induced upregulation of p27 is due to increased stability of p27. To consolidate this finding, we determined the effect of the proteasome inhibitor MG132 on p27 levels in control and DAP-treated cells. Immunoblotting of crude cell lysate demonstrated that MG132 treatment increased p27 levels in control cells, but not DAP-treated cells. However, MG132 treatment increased p21 levels in both control and DAP-treated cells (Figure 2C). These results indicate that DAP upregulates p27 primarily by inhibiting p27 protein degradation.

### Upregulation of p27 by DAP is dependent on p21

**Figure 3:**
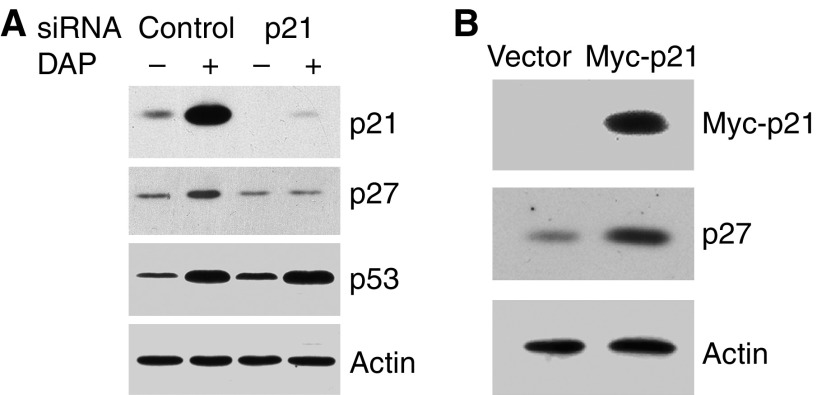
DAP upregulates the expression of p27 in a p21-dependent manner. (**A**) A2780 cells were transfected with control or p21 siRNA for 5 h, and then incubated with normal media for 1 h before being exposed to 0.6 *μ*M DAP or vehicle for another 30 h. Cell lysates were then prepared and analysed by immunoblotting with antibodies specific for p21, p27, p53 and actin. (**B**) A2780 cells were transfected with 2.5 *μ*g of control or *myc*-p21 plasmid for 5 h. After 24 h, lysates of transfected cells were prepared and immunoblotted with anti-p21, anti-p27 and anti-actin antibodies.

To examine if upregulation of p27 results from p21 induction, we first knocked down p21 expression by p21-specific siRNA and determined the effect on p27 induction in response to DAP treatment. Immunoblotting of crude lysates from control siRNA or p21 siRNA-transfected cells showed that siRNA reduced p21 to undetectable levels, with a concomitant small decrease in basal p27 levels (Figure 3A). With DAP, the siRNA abrogated induction of both p21 and p27, whereas it did not prevent p53 induction. As a complimentary approach, we transfected A2780 cells with an expression construct for myc-tagged p21 (myc-p21) to determine whether increasing p21 levels in the absence of DAP treatment is sufficient to upregulate p27. Immnublotting of crude cell lysate from cells transfected with control vector or myc-p21 construct demonstrated that the level of p27 increased by 2- to 3-fold in p21-transfected cells (Figure 3B). These results indicate that p21 induction is responsible for upregulating p27 during DAP-induced G1-phase checkpoint response.

### DAP induces preferential increase of p27 in the CDK2 complex

**Figure 4:**
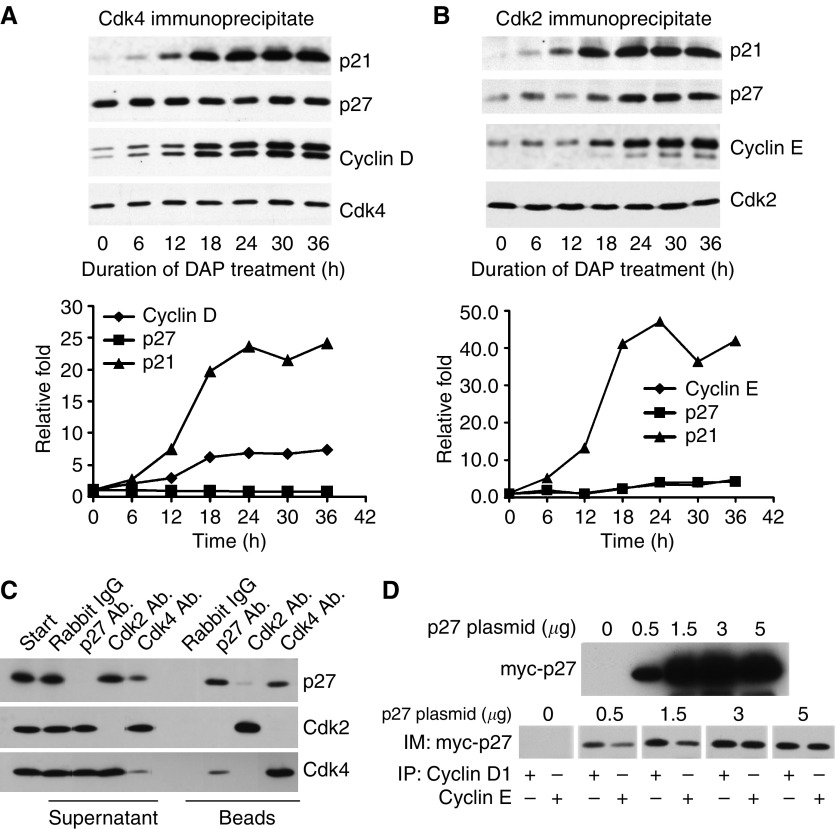
DAP promotes the preferential increase of p27 in CDK2 complex. (**A**) Crude lysates of A2780 cells treated with 0.6 *μ*M DAP for the indicated lengths of time were immunoprecipitated with anti-CDK4 antibody, and the immunocomplex was either immunoblotted with anti-CDK4, anti-cyclin D, anti-p27 or anti-p21 monoclonal antibody (upper panel). The signals from the CDK4, cyclin D, p27 and p21 proteins were quantified by densitometry after immunoblotting. The ratio of cyclin D, p27 or p21 to CDK4 was calculated, normalised against the level at time 0 and plotted against time (lower panel). (**B**) Crude cell lysates of A2780 cells treated with 0.6 *μ*M DAP for the indicated lengths of time were immunoprecipitated with anti-CDK2 antibody, and the immunocomplex was either immunoblotted with anti-CDK2, anti-cyclin E, anti-p27 or anti-p21 monoclonal antibody (upper panel). The signals from the CDK2, cyclin E, p27 and p21 proteins were quantified after immunoblotting. The ratio of cyclin E, p27 or p21 to CDK2 was calculated, normalised against the level at time 0 and plotted against time (lower panel). (**C**) Majority of p27-, CDK2- or CDK4-containing complexes was immunoprecipitated from crude lysates of control A2780 cells with excess anti-p27, CDK2 or CDK4 antibody, respectively. Immunocomplexes or supernatants after immunoprecipitation were immunoblotted with anti-p27, anti-CDK2 and anti-CDK4 antibodies. Rabbit IgG was utilised as a control for these antibodies in the experiment. (**D**) A2780 cells were transfected with 2.5 *μ*g of control or *myc*-p27 plasmid for 5 h. After 24 h, lysates of transfected cells were prepared. Majority of cyclin D- or cyclin E-containing complexes was immunoprecipitated with anti-cyclin D or cyclin E antibody and immunoblotted with anti-myc antibody.

To examine the role of upregulated p27 in DAP-mediated inhibition of G1-phase CDK, we used CDK4 or CDK2 antibodies to immunoprecipitate CDK complexes from crude lysates of cells collected over a 36-h treatment period with DAP, and then determined the levels of both p21 and p27 in the two G1-phase CDK complexes. As shown in Figure 4A and B, the level of p21 in both CDK4 and CDK2 complexes increased from 6 to 24 h, which correlated with the increase in the amount of p21 in crude cell lysates (see Figure 1B). In contrast, the level of p27 increased progressively only in the CDK2 complex from 24 to 36 h, which coincided with the increase in p27 in crude cell lysates (cf. Figure 4B and 1B). Increases in cyclin D and E paralleled those of p21 and p27, and may reflect the role of these inhibitors in the assembly of CDK complexes.

The results above appear to indicate that DAP treatment selectively increases p27 within the CDK2 complex, but not CDK4 complex. However, based on literature reports, the CDK4 complex, rather than the CDK2 complex, is the preferential binding partner of p27 in normal cell lines (Toyoshima and Hunter, 1994; Poon *et al*, 1995). In order to determine whether this was also the case in the A2780 cancer cell line, p27 was immunoblotted in total CDK4 and CDK2 complexes isolated by immunoprecipitation from untreated cells. The results in Figure 4C indicate that immunoprecipitation with the p27 or CDK4 antibody co-immunoprecipitated all or most of this inhibitor as assessed by concomitant depletion in the supernatant, whereas the CDK2 antibody co-immunoprecipitated only negligible amounts of p27. These findings indicate that untreated A2780 cells also have most of the p27 bound to CDK4, but not CDK2. On the other hand, not all of the CDK4 complex was associated with p27 as immunoprecipitation of p27 in the supernatant only partially co-immunoprecipitated CDK4. Similarly, most of the CDK4 and CDK2 complex was free of p21 (data not shown).

To further examine the specificity of p27 binding, we transfected A2780 cells with different amounts of myc-p27 expression vector and investigated the distribution of ectopically expressed p27 between the two CDKs. As shown in Figure 4D, myc-p27 was evenly distributed between total cyclin D (representing the CDK4 complex) and cyclin E (representing the CDK2 complex) immunoprecipitates when myc-p27 expression was high, whereas distribution favoured CDK4/cyclin D complex at lower expressions. The results indicate that in control tumour cells, both endogenous and exogenous p27 are associated with the CDK4 complex to a greater extent. In contrast, DAP treatment promotes the specific increase of p27 in the CDK2 complex even though substantial levels of CDK4 are available for potential binding to this inhibitor. It is possible that interaction of p27 with another protein could alter its preference from CDK4 to CDK2. In this regard, reports have suggested that cellular increase in p27 may lead to its increased interaction with the modulator protein Spy1 and, thereby increase incorporation of p27 and Spy1 into the CDK2 complex (Porter *et al*, 2003).

### DAP treatment does not recruit additional component(s) into CDK complexes

**Figure 5:**
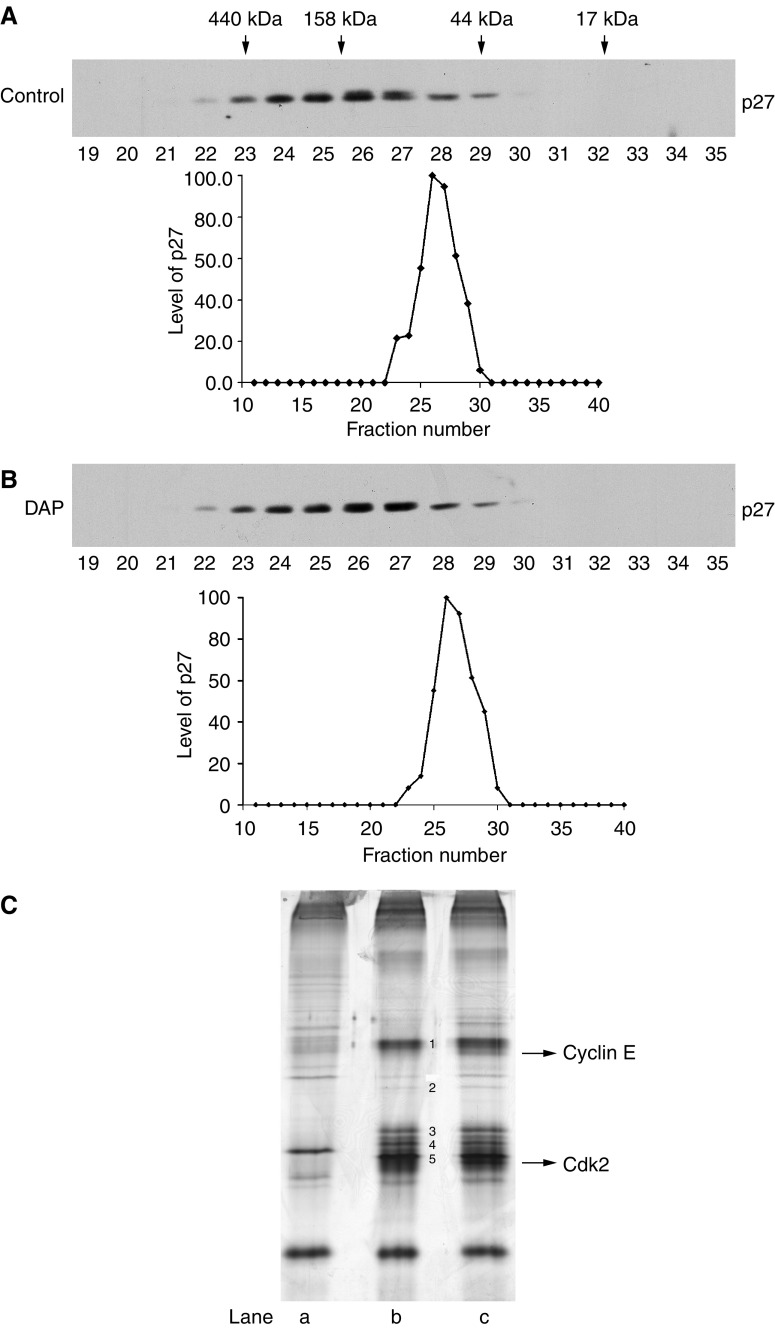
DAP treatment does not recruit new component(s) into p27-containing complexes. (**A**) Crude cell lysates prepared from untreated cells were fractionated on a 40-ml gel filtration AcA34 column. Proteins from fractions 19–35 were separated by SDS–PAGE, electrophoretically blotted onto nitrocellulose membranes, and immunoblotted with anti-p27 antibody. (**B**) Crude cell lysates made from DAP-treated (0.6 *μ*M; 24 h) cells were fractionated by gel filtration and immunoblotted with anti-p27 antibody, as described above in (**A**). (**C**) Large-scale immunoprecipitation of untreated or DAP-treated (0.6 *μ*M; 24 h) A2780 cell lysates was performed with covalently linked anti-p27 or control rabbit IgG; eluted polypeptides from antibody beads were then resolved by SDS–PAGE and visualised by silver staining. CDK2 and cyclin E immunoprecipitated by anti-p27 antibody following DAP treatment were identified by mass spectrometry. Lane a: Rabbit IgG immunoprecipitates of untreated cell lysates. Lane b: Anti-p27 immunoprecipitates of untreated cell lysates. Lane c: Anti-p27 immunoprecipitates of DAP-treated cell lysates.

To explore the possibility that DAP-mediated increase in p27 recruitment within the CDK2 complex, rather CDK4 complex, was due to binding of a modulator protein, we measured the apparent molecular size of p27-containing complexes from untreated and DAP-treated cells by gel filtration and determined whether p27-containing complexes underwent a size shift in response to DAP treatment. Calibration of the column with molecular weight standards showed that this column had a linear separation range of 17–440 kDa (data not shown). Western blot analysis of p27 in eluted fractions no. 19–35 showed that p27 complexes from both control and DAP-treated cell lysates peaked in fractions 26 and 27, between the 158- and 44-kDa gel filtration standards, suggesting that no additional component(s) is associated with p27 complexes after DAP treatment (Figure 5A and B). To confirm this, we performed large-scale immunoprecipitation with p27 antibodies from untreated cells or cells treated with DAP for 30 h. The precipitated proteins were then resolved by SDS–PAGE, visualised by silver staining, and unique bands following DAP treatment identified by mass spectrometry. Figure 5C shows result from a representative p27 immunoprecipitation experiment. Compared to the control IgG immunoprecipitate (lane a), the p27 immunoprecipitate from control cell lysate contained five major bands and several minor bands (lane b). These bands were also present at almost similar intensities in the p27 immunoprecipitate from DAP-treated cell lysate (lane c). Interestingly, the p27 antibody precipitated only two unique bands from DAP-treated cell lysate; one migrated at about 50 kDa and the other at about 32 kDa (lane c). By mass spectrometry, they were identified as cyclin E and CDK2, respectively, confirming our observations that DAP treatment effectively increases the amount of p27 in the CDK2/cyclin E complex (see Figure 4B). Taken together, the data from gel filtration chromatography and large-scale immunoprecipitation of p27 did not reveal any additional components that may explain increased binding of p27 to the CDK2 complex after DAP treatment.

### Upregulated p27 inhibits CDK2 kinase activities

**Figure 6:**
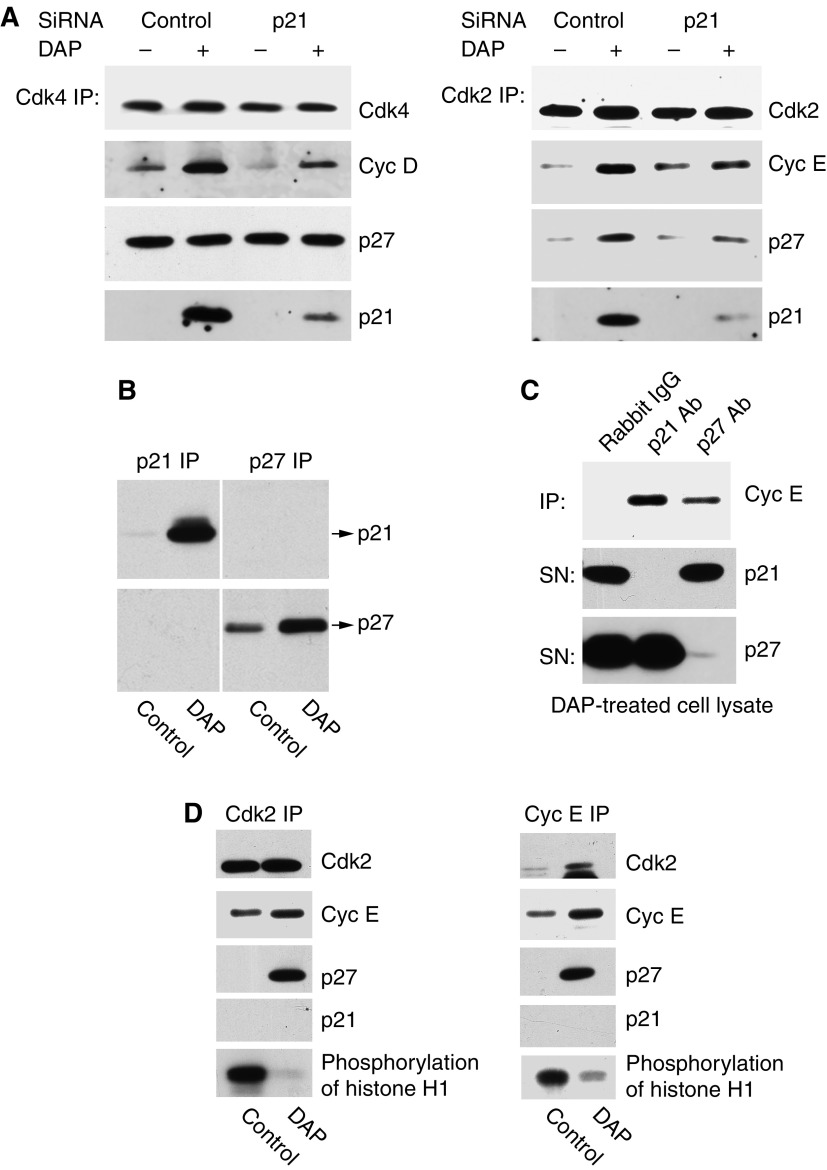
Induction of p21 by DAP stabilises p27 protein in CDK2 complex to inhibit its kinase activity. (**A**) As described in Figure 3A, lysates were prepared from cells exposed to p21 siRNA and 0.6 *μ*M DAP, and then immunoprecipitated with anti-CDK4 or CDK2 antibody. The immunoprecipitates were separated by SDS–PAGE and immunoblotted with antibody specific to p21, p27, CDK4, CDK2, cyclin D1 or cyclin E. (**B**) Crude cell lysates made from control or DAP-treated (0.6 *μ*M; 30 h) A2780 cells were immunoprecipitated with either anti-p21 or anti-p27 antibodies, and the precipitated proteins were immunoblotted with anti-p21 or anti-p27 antibody. (**C**) Majority of p21- or p27-containing complexes was immunoprecipitated from DAP-treated (0.6 *μ*M; 30 h) A2780 cell lysates with excess anti-p21 or anti-p27 antibody. The immunoprecipitate (IP) was separated by SDS–PAGE and immunoblotted with anti-cyclin E antibody. Supernatants (SN) were immunoblotted with anti-p21 or anti-p27 antibody to ensure respective immunoprecipitation was quantitative. (**D**) CDK2 complexes were immunoprecipitated with anti-CDK2 (left panel) or anti-cyclin E (right panel) antibodies from either control A2780 cell lysate, which had been depleted of p21 and p27-containing complexes with respective antibodies (left lane), or DAP-treated (0.6 *μ*M; 30 h) cell lysate, in which only the p21-containing complexes had been immunodepleted (right lane). CDK2, cyclin E, p27 and p21 in these immunoprecipitates were subjected to immunoblot analysis, and CDK2 activities were determined as described in Experimental Procedures.

As Figure 3 demonstrated that DAP-induced p21 is responsible for the increase in the total level of p27, it was necessary to examine whether p21 was also required for increasing p27 in the CDK2 complex following DAP treatment, and if this resulted in p27-dependent inhibition of CDK2 kinase activity. To explore this possibility, we prevented DAP-mediated induction of p21 by siRNA and determined the effect on p27 levels in CDK4 and CDK2 complexes. Immunoblots of p27 in CDK4 immunoprecipitates showed that p21 siRNA did not affect the level of p27 in CDK4 complex following DAP treatment (Figure 6A). In contrast, the high level of p27 in CDK2 complex in DAP-treated cells was substantially suppressed by knockdown of p21 expression, indicating that the increase of p27 in CDK2 complex is dependent on p21. However, whether the induced p21 regulated p27 levels in CDK2 by direct binding or through an indirect mechanism needed to be resolved. Therefore, we performed reciprocal immunoprecipitation with p21 or p27 antibody and examined whether p21 and p27 co-existed within the immunoprecipiate from control or DAP-treated cells. As shown in Figure 6B, p21 and p27 did not physically interact, and, therefore, did not co-exist within the same CDK complex in untreated or DAP-treated cell lysate. This excluded the possibility that p21 regulates p27 through direct binding.

Although our data show that DAP-induced p21 is responsible for increasing p27 in CDK2 complex, the functional consequence of this required examination. Therefore, we immunoprecipitated majority of p21- or p27-bound CDK2 complexes with excessive levels of p21 or p27 antibodies and then measured the relative amounts of CDK2 complex to which they were bound in cells exposed to DAP. As expected from Figure 6B, immunoprecipitation of p21 or p27 from DAP-treated cell lysates substantially depleted the inhibitor from the cell lysate, but did not co-deplete the second inhibitor (Figure 6C). Immunoblots and densitometry of cyclin E in immunoprecipitates indicated that the level of CDK2/cyclin E complex associated with p21 was about three times higher than with p27 (Figure 6C). Previously, we have reported that most of the CDK2 complexes contained p21 and p27 after DAP treatment (He *et al*, 2005), but in the present study, we demonstrate that p21 and p27 do not co-exist. Taken together, the results in Figure 6C indicate that p21 is bound to about 75% CDK2/cyclin E complex and p27 to the remaining 25%. We have previously demonstrated that this p21 binding dramatically decreases CDK4 or CDK2 activities of the CDK4 or CDK2 immunoprecipitate after p27 was immunodepleted (He *et al*, 2005). Therefore, in the present study, it was of interest to examine if p27-containing CDK2 complex was also inactive. For this purpose, we immunoprecipitated, using either CDK2 (Figure 6D, left panel) or cyclin E (right panel) antibodies, CDK2 complexes from p21- and p27-depleted control cell lysates or p27-associated CDK2 complex from p21-depleted DAP cell lysates. As shown in Figure 6D, CDK2/cyclin E complex lacking p27 was active (left lane), whereas that associated with p27 was inactive (right lane). This demonstrates that p27 effectively inhibits the kinase activity of the CDK2/cyclin E complex.

In summary, these results demonstrate that p27 levels in the CDK2 complex is regulated by DAP-induced p21, and stabilisation of p27 within the complex inhibits CDK2 activity.

## DISCUSSION

Our present study demonstrates that p21 not only inhibits CDK activity directly, but also indirectly through stabilising p27 protein that increases cellular levels of this inhibitor. Moreover, DAP-induced p21 promotes the selective increase of p27 in CDK2 complexes, and not in CDK4 complexes, and this selective increase contributes to the inhibition of CDK2 kinase activity. Thus, our data provide evidence that p27 contributes to DAP-induced G1-phase checkpoint response in a manner similar to that observed with p21. This is the first evidence that p27 regulation is linked directly to activation of the p53/p21 pathway by a DNA-damaging agent.

Hyperproliferation of cancer cells is associated with deregulation of cell cycle progression, which is driven by the activities of CDKs. Therefore, inhibition of CDK activity is a realistic goal in the clinical management of cancer (McDonald III and El Deiry, 2000; Malumbres and Carnero, 2003). This is particularly evident from studies with selective small molecule CDK inhibitors, which halt G1/S transition and induce cell death (Senderowicz, 2003). The vital contribution of DAP-induced p27 in inhibiting CDK2 in much the same way likely contributes to the cellular effects of this novel platinum agent. This is consistent with the acknowledged significance of p27 in cancer progression and antitumour drug response, and stems from the reported correlation between low p27 expression and either high tumour grades or poor survival following chemotherapy treatment (Sgambato *et al*, 2000; Nho and Sheaff, 2003). Conversely, its overexpression in cancer cells inhibits tumour cell growth by enforcing cell cycle arrest and apoptosis, and this has contributed to p27 being referred to as a tumour suppressor (Katayose *et al*, 1997; Wang *et al*, 1997). Likewise, p21 is also now becoming recognised as a tumour suppressor (Brugarolas *et al*, 1998; Martin-Caballero *et al*, 2001), and this is in concordance with its role in facilitating cisplatin-induced cell death when overexpressed ectopically (Lincet *et al*, 2000; Qin and Ng, 2001, 2002). In consonant with this, deletion or depletion of p21 inhibits apoptosis and induces resistance (Hsu *et al*, 1999), which is in agreement with the reported failure of cisplatin to upregulate p21 in cisplatin-resistant tumour cells (Mujoo *et al*, 2003, 2005). In contrast, several reports have provided evidence that p21 protects cells from apoptosis (Gartel and Tyner, 2002; Liu *et al*, 2003), and this makes it likely that cell context and the cellular signalling pathway induced by the specific chemotherapeutic drug determine whether p21 facilitates or antagonises apoptosis. With DAP, however, expression of p21 is crucial for CDK inhibition and to prevent cell proliferation (Kuang *et al*, 2001; He *et al*, 2005), and it is likely from the present studies that these effects are mediated in part through p21-dependent stabilisation of p27 within the CDK2 complex.

Regulation of p27 in human cancers is predominantly through a mechanism involving Skp2-mediated proteosomal degradation of p27 (Bloom and Pagano, 2003). Furthermore, CDK2/cyclin E-dependent phosphorylation of p27 at Thr-187 has been suggested as essential for the recognition of p27 by Skp2. Therefore, downregulation of Skp2 level or inhibition of proteasomal activity, as in this study with MG132, increases p27 level. As we have demonstrated that increases in p27 by DAP are dependent on p21, the observed p21-mediated inhibition of CDK2 activity is the likely mechanism, with resultant decrease in phosphorylation of p27. Although previous reports have suggested that p27 upregulation may involve p21 (Liu *et al*, 2000; Steinman *et al*, 2001; Munoz-Alonso *et al*, 2005), the present study not only provides definitive evidence for this, but also demonstrates for the first time that this upregulation is robust, inhibits CDK2/cyclin E and occurs in response to DNA damage.

Unlike previous reports on the induction of p27 by ionising radiation or antitumour agents (Vikhanskaya *et al*, 1996; Balasubramanian *et al*, 1999; Vivo *et al*, 2003; Zhou *et al*, 2004), we have examined not only the overall upregulation of p27 by DAP, but also its increase specifically within the CDK2 complex to better understand the contribution to CDK2 inhibition. Our data provide direct evidence that p27 is responsible for ∼25% of the inhibited CDK2, with the remaining inhibition attributable to p21. The relatively lower contribution of p27 is surprising, considering that the absolute amount of p27 in the cell after DAP treatment is similar to or greater than that of p21. This inconsistency may be explained by the possibility that either the stoichiometry of p27:CDK2 is greater than that for p21:CDK2 in the inhibited complex, or that a portion of the induced p27 has functions other than in CDK inhibition. Although it is not clear from these studies whether there is sufficient induction of p21 to inhibit all CDK2 molecules if p27 were absent, it is overtly apparent that DAP-induced p27, like p21, contributes significantly to the inhibition of CDK2 activity. On the other hand, as p27 levels are not altered in the CDK4 complex, and p27 and p21 do not co-exist within the same complex, it is likely that p27-free CDK4 complexes are inhibited predominantly through recruitment of DAP-induced p21.

**Figure 7:**
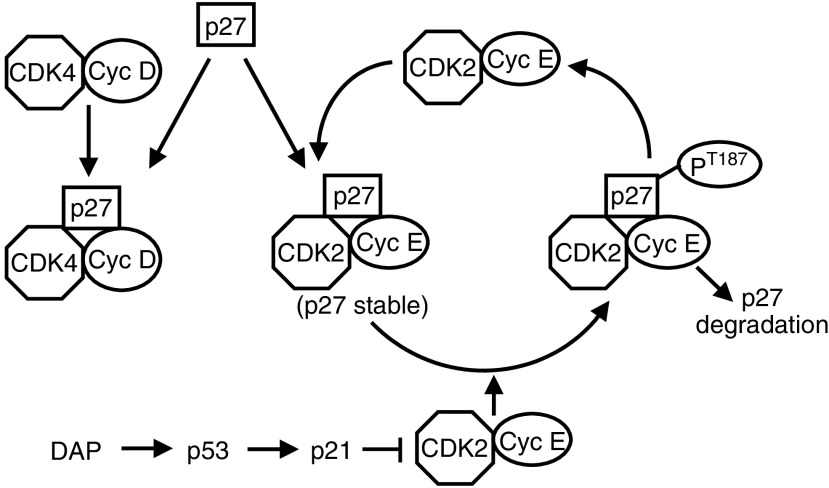
Proposed model for p21-dependent regulation of p27 following DAP treatment.

Our study has also brought awareness to the novel observation that p21-dependent increases of p27 are only reflected within the CDK2 complex, and not CDK4 complex. With the data available, it is possible to propose a conceptual model to explain the induction of p27 by DAP. In this model, basal levels of p27, which are regulated independently of p21, are recruited into both CDK4 and CDK2 complexes (Figure 7). However, the recruitment of p27 into the CDK2 complex results in immediate phosphorylation of the inhibitor by CDK2 activity that leads to rapid degradation of p27 via the ubiquitin pathway. Based on literature evidence, the p27 bound to CDK2 does not undergo intramolecular phosphorylation, but it is phosphorylated by the active CDK2 complex that is free of p27 (Montagnoli *et al*, 1999; Xu *et al*, 1999). Consequently, the p27 associated with CDK2 is kept to a relatively low level. Once p21 is induced, it inhibits CDK2 and, thereby prevents p27 phosphorylation; this stabilises and increases the level of p27 within the CDK2 complex, which then becomes inhibited. Based on such a model, CDK2 inhibition will need to reach a critical threshold level before p27 within the CDK2 complex is stabilised, and this may well explain the observed three-fold greater contribution to CDK2 inhibition of p21 than p27. In contrast, the p27 associated with CDK4 is not susceptible to phosphorylation by CDK2, and so the inhibitor remains in a stabilised form within the CDK4 complex (Sheaff *et al*, 1997; Vlach *et al*, 1997). This is supported by our results with p21-siRNA, which maintains CDK2 in an active state but does not reduce to any degree the level of p27 bound to the CDK4 complex.

In conclusion, our data highlight the significance of p21 and p27 expression in DAP-mediated G1-phase cell cycle arrest in order to prevent G1/S transition. More importantly, we have provided evidence that increased association of p27 within the CDK2 complex is mediated through a p21-dependent pathway, which prevents destabilising phosphorylation of p27. We have previously demonstrated the importance of the p53/p21 pathway in cell cycle effects of DAP. Based on our present study, which demonstrates substantial p27 induction and p27-dependent inhibition of CDK2 by DAP, it is appropriate for the p53/p21/p27 axis to assume greater prominence and become a new focus of attention in checkpoint response to DNA damage.
